# Supplementary figures and images for: Dongsha Atoll is an important stepping-stone that promotes regional genetic connectivity in the South China Sea
Source: PeerJ. 2021 Aug 31;9:e12063. doi: 10.7717/peerj.12063 (PMC8415289; doi:10.7717/peerj.12063)

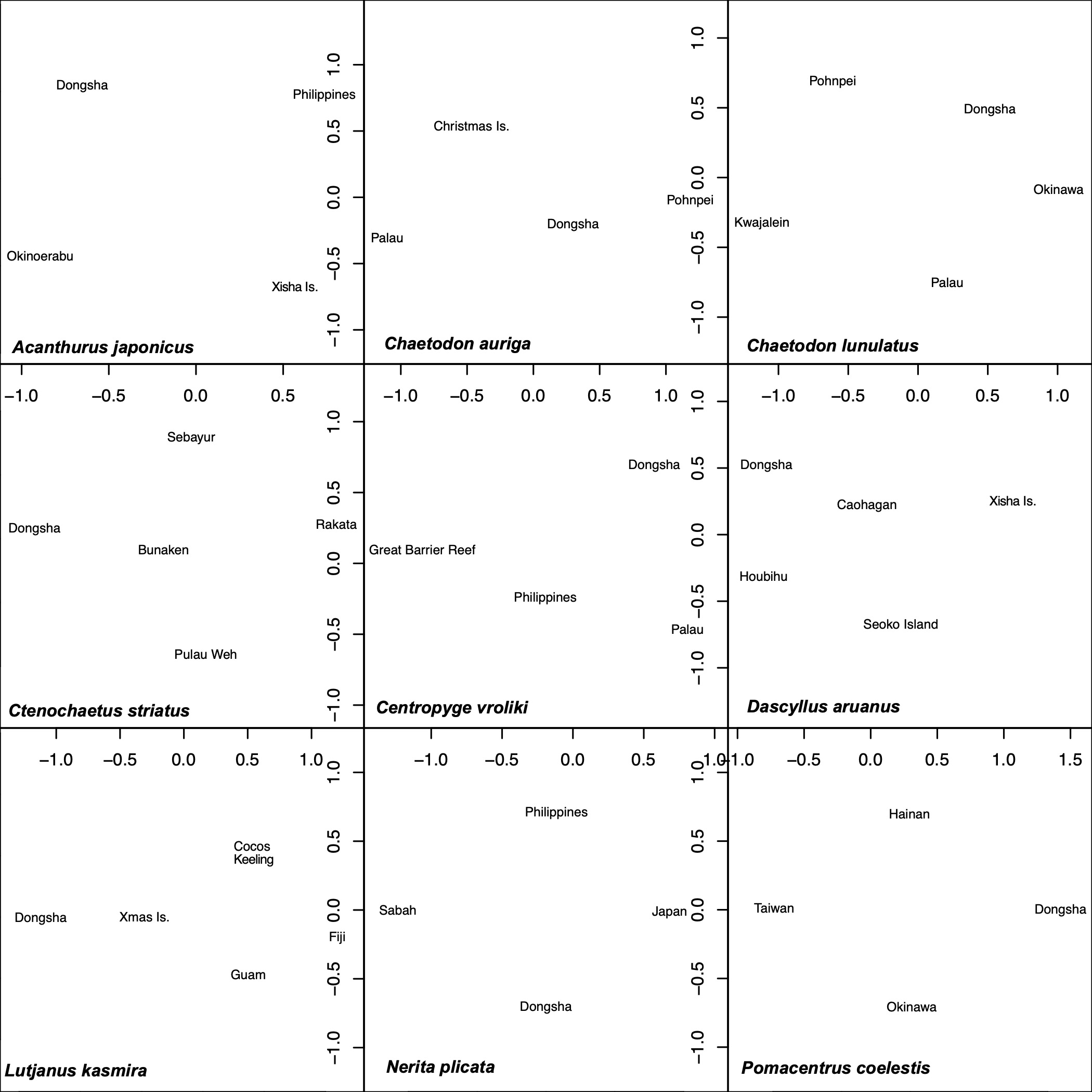

Supplement: Supplemental Information 2 — Non-metric dimensional scaling (NMDS) results based on FST matrices in Fig. S2 depict Dongsha’s genetic relationship with neighboring populations in two-dimensional space for all nine species. [file peerj-09-12063-s002.jpg]
